# Extreme value modelling of Ghana stock exchange index

**DOI:** 10.1186/s40064-015-1306-y

**Published:** 2015-11-12

**Authors:** Ezekiel N. N. Nortey, Kwabena Asare, Felix Okoe Mettle

**Affiliations:** Department of Statistics, University of Ghana, Box LG 115, Legon, Ghana

**Keywords:** Extreme value theory (EVT), Peak Over Threshold (POT), Generalized Pareto Distribution, Value at risk (VaR), Expected shortfall

## Abstract

Modelling of extreme events has always been of interest in fields such as hydrology and meteorology. However, after the recent global financial crises, appropriate models for modelling of such rare events leading to these crises have become quite essential in the finance and risk management fields. This paper models the extreme values of the Ghana stock exchange all-shares index (2000–2010) by applying the extreme value theory (EVT) to fit a model to the tails of the daily stock returns data. A conditional approach of the EVT was preferred and hence an ARMA-GARCH model was fitted to the data to correct for the effects of autocorrelation and conditional heteroscedastic terms present in the returns series, before the EVT method was applied. The Peak Over Threshold approach of the EVT, which fits a Generalized Pareto Distribution (GPD) model to excesses above a certain selected threshold, was employed. Maximum likelihood estimates of the model parameters were obtained and the model’s goodness of fit was assessed graphically using Q–Q, P–P and density plots. The findings indicate that the GPD provides an adequate fit to the data of excesses. The size of the extreme daily Ghanaian stock market movements were then computed using the value at risk and expected shortfall risk measures at some high quantiles, based on the fitted GPD model.

## Background

Recently, an issue of concern to most risk managers and financial analysts are the events that occur under certain extreme market conditions. This refers to events which have the tendency to produce huge and unexpected losses that could affect and also probably lead to bankruptcies and consequently, systemic risk (Gavril 2009). Hence, the extreme value theory (EVT), traditionally used in fields like hydrology and meteorology has in the last years been successfully incorporated into financial risk modelling. Ren and Giles (2007) described EVT as a theory for assessing the asymptotic probability of extreme values, further expanding that the theory models the tail part of the distribution where the risk exists. The two main methods of the EVT approach are the Peak Over Threshold (POT) and the Block Maxima methods. The POT method is preferred in this paper since it has been proven empirically to efficiently utilize more of the data and hence produce more reliable findings compared to the Block Maxima approach (McNeil and Frey 2000; Matthys and Beirlant 2000; Coles 2001; Blum et al. 2002; Gilli and Kellezi 2006).

What makes the EVT very appealing is the fact that the nature of the asymptotic distribution does not necessarily depend on the exact distribution of returns. DuMouchel (1983) succinctly expressed these benefits of employing EVT to financial risk management in the statement: “Letting the tails speak for themselves”. This characteristic is particularly appealing as risk managers are primarily concerned with avoiding big unexpected losses and sudden crashes rather than long sequences of medium-sized losses. This is mainly due to the empirical observation that the final position of a portfolio is more affected by a few extreme movements in the market rather than by the sum of many small movements (Rocco 2011).

As the estimation of these rare events involve the estimation of extreme quantiles, risk measures such as the value at risk (VaR) and expected shortfall (ES) have been found to be more appropriate compared to the others which rely on the entire distribution since they capture the quantile risks in the tails of the distribution (Harlow 1991).

This paper therefore models the Ghana all-shares stock index using the EVT method and further computes the risk measures associated with the Ghanaian stock market, under the EVT framework.

There have been very few studies investigating the tail behaviour of the returns of the Ghana Stock Exchange indices and also computing the resulting market risks using the VaR or the expected shortfall approaches.

The main motivation behind this paper is the need to examine the performance of the EVT method in the analysis of the Ghanaian stock market. This paper thus contributes to empirical evidence of the research into the behaviour of the extreme returns of financial series in Africa and specifically in Ghana.

The rest of the paper is organized as follows: “Method” details the EVT methodology and the VaR and ES risk measures, Data discusses the Ghana stock market and the data employed, “Results and discussions” empirically examines the fitness of the EVT and results from the two risk measures, “Conclusion” concludes the paper.

## Method

The Peak over Threshold approach (POT) of the EVT is described at this stage. For a set of observations $$ X_{1} ,X_{2} ,X_{3} , \ldots ,X_{n} $$ with cumulative distribution function *F*(*x*), and a predetermined threshold *u*, the interest here is in that of the distribution of the exceedances or the values of *x* above the threshold *u*, given that *u* is in fact exceeded. Thus an exceedance occurs when $$ X_{i} > u $$, for any $$ i = 1,2, \ldots ,n $$. Hence we can define $$ y = X_{i} - u $$ and its corresponding distribution function is known as the conditional excess distribution function $$ F_{u} (y) $$ defined as $$ F_{u} (y) = P(x - u \le y/x > u), $$$$ 0 \le y \le x_{F} - u $$, where $$ x_{F} < \infty $$ is the right endpoint of *F.*

1 $$ \begin{aligned} F_{u} (y) = P(x \le y + u/x > u)& = \frac{P(x \le y + u,x > u)}{P(x > u)} \hfill \\ & = \frac{P(x \le y + u) - P(x \le u)}{P(x > u)} \hfill \\ \end{aligned} $$

Hence, since $x = u + y$ for $X > u$, expressing $F$ in terms of $F_{u}$ gives

2 $$ F_{u} (y) = \frac{F(u + y) - F(u)}{1 - F(u)} = \frac{F(x) - F(u)}{1 - F(u)} $$

Clearly, as the bulk of the observations lie in the area 0–*u,* the estimation of its distribution is quite straightforward. However, estimation of the portion above *u* with distribution $F_{u}$ proves problematic since only a few observations are present in this range. The estimation of this conditional excess distribution function was proposed in the following theorem.

Theorem (Pickands 1975; Balkema and de Haan 1974): If the underlying distribution of the returns series belongs to the maximum domain of attraction (MDA) of the Generalised Extreme Value (GEV) distribution, as the threshold *u* becomes large, the distribution function of the exceedances over the threshold has approximately a Generalized Pareto Distribution (GPD).

Hence, for a large class of underlying distribution functions *F*, and also a predetermined high threshold *u,* the conditional excess distribution function $F_{u} (y)$ is very well approximated by

$$ F_{u} (y) \approx G_{\xi ,\sigma } (y),\quad u \to \infty $$

where

3 $$ G_{\xi ,\sigma } (y) = \left\{ \begin{array}{l} {{1 - \left( {1 + \frac{\xi }{\sigma }y} \right)^{{ - \frac{1}{\xi }}} ,  \xi \ne 0}} \\ {{1 - \exp \left( {\frac{ - y}{\sigma }} \right), \quad   \xi = 0}} \end{array} \right.\;\quad {for} \,\,{y} \in \left\{\begin{array}{l} {{\left[ {0,(x_{F} - u)} \right],\quad \xi \ge 0}} \\ {{\left[ {0, - \frac{\sigma }{\xi }} \right],  \quad \xi < 0}} \end{array} \right. $$

$G_{\xi ,\sigma } (y)$ is referred to as the Generalized Pareto Distribution (GPD) with shape parameter also known as the tail index $\xi$ and a scale parameter $\sigma$. Thus, the value of the scale parameter $\sigma$ shows how heavy the tail of the distribution is with a large value indicating a very heavy tail and hence the more spread out the distribution. Gilli and Kellezi (2006) indicated that generally, an upper tail for financial losses can’t be fixed and because of this, only distributions with shape parameter $\xi \ge 0$ are suited to model financial return series.

Also, it is possible to express the GPD as a function of *x* by defining $x = u + y$. In which case we have Eq. (4) as

4 $$ G_{{\xi ,\sigma }} (x) = \left\{ {_{{1 - \exp \left( {{\hbox{${ - (x
- u)}$} \!\mathord{\left/ {\vphantom {{ - (x - u)} \sigma }}\right.}
\! \hbox{$\sigma $}}} \right) \quad if \quad \xi  = 0}}^{{1 - \left(
{1 + \frac{{\xi (x - u)}}{\sigma }} \right)^{{{\hbox{${ - 1}$} \hbox{$\xi $}}}} \quad \quad
if  \quad  \xi  = 0}} \quad {x} \in \left\{ {_{{\left[ {u,\;u -
{\sigma   \xi }} \right] \quad\,\, if \quad \xi \,\, <\,\,
0}}^{{\left[ {u,\;\infty } \right] \quad \quad \,\,\,\,\,if \quad
\xi \,\ \ge \,\,0 }} } \right.} \right. $$

If we set *u* = 0 and $$ \sigma = 1 $$, the resulting equation is known as the standard Generalized Pareto Distribution (GPD).

Usually, attention is restricted to the study of the shape parameter $$ \xi $$ since it proves more crucial. When $$ \xi > 0, $$ the tail of the GPD is of the Pareto type and when $$ \xi = 0, $$ the tail is of the exponential type. Finally, when $$ \xi < 0, $$ the GPD has a finite right endpoint. The two approaches mostly employed in the parameter estimation of the GPD are the Maximum Likelihood Estimation (MLE) method and the method of Probability Weighted Moments (PWM). These methods are therefore considered in this paper.

In a bid to estimate the market risks associated with holding financial positions on the Ghana stock market, the VaR and expected shortfall (ES) frameworks are considered.

The VaR of a financial portfolio at a confidence level, $$ 0 < p < 1, $$ can be defined as the smallest number $$ l $$ such that the probability of a loss L exceeding $$ l $$ over a certain time horizon is smaller than or equal to $$ (1 - p). $$ Thus it is given as

5 $$ VaR_{p} = \inf \left\{ {l \in R/P(L > l) \le (1 - p)} \right\}. $$

Alternatively, if a random variable *X* models the negative returns on a certain financial portfolio with a corresponding cdf *F,* then the VaR at the *p*-*th* quantile is given by

6 $$ VaR_{p} = F^{ - 1} (1 - p),\quad 0 < p < 1, $$

where $$ F^{ - 1} $$ known as the quantile function is the inverse of the cdf *F*.

For a set of observations $$ X_{1} ,X_{2} ,X_{3} , \ldots ,X_{n} $$ with cdf *F*(*x*), and a predetermined threshold *u*, the conditional excess distribution function $$ F_{u} (y) $$ has already been established as

7 $$ F_{u} (y) = \frac{F(x) - F(u)}{1 - F(u)} $$

Hence, with some fairly simple algebra, the form of *F(x)* can be written as

8 $$ F(x) = (1 - F(u))Fu(y) + F(u) $$

Given *n* as the total number of observations and $$ Nu $$ as the number of observations above the threshold *u,* the expression $$ F(u) $$ can be estimated by $$ \frac{{\left( {n - Nu} \right)}}{n}. $$

Also, the expression for the GPD written in terms of *x* = *y* + *u* is given by

9 $$G\xi ,\sigma (x) = 1 - \left( {1 + \xi \left(\frac{x - u}{\sigma
}\right)} \right)^{^{{{ - 1} / \xi }} } $$

Therefore, the tail estimate can be written as

10 $$ \hat{F}(x) = \frac{Nu}{n}(1 - G\xi ,\sigma (x)) + \left( {1 - \frac{Nu}{n}} \right) = 1 - \frac{Nu}{n}\left( {1 + \frac{\xi }{\sigma }(x - u)} \right)^{{ - 1} / \xi } $$

The estimate of the VaR with a given probability *p* under the GPD approach is obtained by inverting expression above to obtain the inverse function of $$ \hat{F}(x) $$ or the quantile function and is given as

11 $$ VaR_p = u + \frac{{\hat{\sigma }}}{{\hat{\xi }}}\left( {\frac{n}{Nu}(1 - p)^{{ - \hat{\xi }}} - 1} \right) $$

The expected shortfall can be described as the expected value of the size of the loss exceeding the VaR with some level of probability on the condition that the loss actually exceeds the VaR. It can thus be expressed as

12 $$ ESp = E(X/X > VaR_p),\quad 0 < p < 1 $$

This expression can further be written as

13 $$ ES_p = VaR_p + E\left( {X - VaR_p/X > VaR_p} \right) $$

From the definition of the mean excess function of the GPD with distribution function F and some high threshold u, the expected shortfall can similarly be described by considering the $$ VaRp $$ as the threshold level.

Recall that if X follows a GPD with threshold *u*, the mean excess function is given by

14 $$ \begin{aligned} e(u) &= E\left( {X - u/X > u} \right) \quad {\text{ie}}. \quad X - u/X > u \sim GPD(\xi ,\sigma ) \hfill \\ \quad \quad &= \frac{\sigma + \xi u}{1 - \xi },\quad \sigma + \xi u > 0 \hfill \\ \end{aligned} $$

Note that for a high threshold value $$ VaR_p > u $$, the excess function can be written as

15 $$ X - VaR_p/X > VaR_p = (X - u) - (VaR_p - u)/(X - u) > (VaR_p - u) $$

Hence it can be shown that $$ X - VaR_p/X > VaR_p \sim GPD(\xi ,\sigma + \xi (VaR_p - u)). $$

The expected Shortfall under the GPD framework is therefore estimated by

16 $$ ES_p = VaR_p + \frac{{\hat{\sigma } + \hat{\xi }(VaR_p - u)}}{{1 - \hat{\xi }}} $$

which can also be expressed as

17 $$ ES_p = \frac{VaR_p}{{1 - \hat{\xi }}} + \frac{{\hat{\sigma } - \hat{\xi }u}}{{1 - \hat{\xi }}} $$

## Data

The study employed secondary data obtained from the Ghana Stock Exchange (GSE). It consists of 2226 daily closing prices of GSE all-shares index spanning the years 2000–2010. This index is computed by the GSE based on the values of stocks of each of the companies listed on the stock exchange. Thus it represents a measure of the overall performance of the stock market. The GSE was incorporated in July 1989 and is presently the principal stock exchange of Ghana. It currently has 40 equity listings from 35 companies, mostly Ghanaian, 1 corporate bond, 3 government bonds, and 1 preference share. The exchange is dominated by the manufacturing and brewing industries, followed by the banking sector. The other listings are in the mining, insurance and petroleum sectors. The GSE index was recognised in 1993 as the sixth best performing index among emerging stock markets, with an appreciation in capital by 116 %. By gaining 124.3 % in its index level in 1994, it became the best performing market among all emerging stock markets (GSE 1995). The market capitalization of the GSE was about US$11.2 billion in 2006, US$13.2 billion as at December 2007, and US$ 15.5 billion in 2008. Thus an appreciation of 31.84 % in 2007 (GSE 2008).

Furthermore, as indicated by (UN 1999), an important factor characterising African markets is a very low correlation existing between the African stock markets and the major world stock markets and also between the African markets themselves. The Ghana stock market is no different and is therefore hardly affected by movements on the international stock markets. This makes analysis performed on the stock index very peculiar to the Ghanaian market.

Also, Benimadhu (2003) revealed that a low level of liquidity was among the most prevailing issues affecting African stock markets. In the Ghanaian case, there are very few listed companies even though the stock market has performed well over the years. In addition, investors usually have to wait for a long time to sell out since movement in and out of the market is very low. Furthermore, over the years, there are on average about just 12 listed companies which have been consistent in the market and thus drive the market. This has limited investment options, consequently reduced public participation, and thus reducing the level of liquidity in the market.

**Fig. 1:**
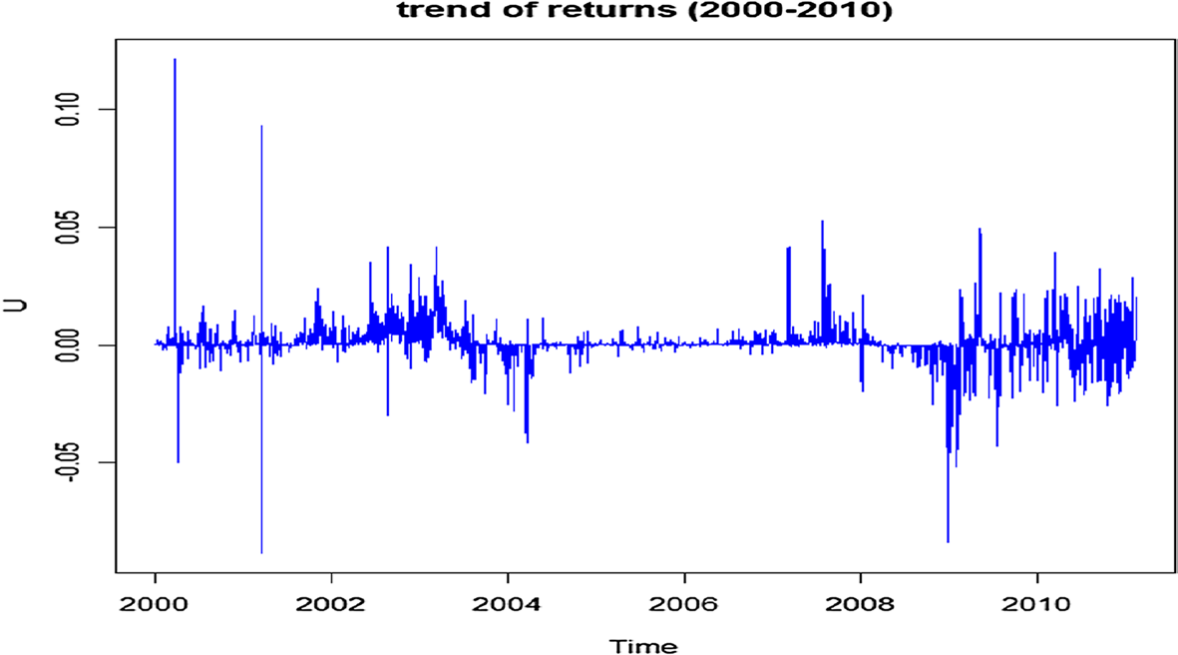
Logarithm returns of daily GSE all-shares index. GSE (2000–2010)

It was observed in 2004 that with the listing of Ashanti Goldfields Company (AGC) now AngloGold Ashanti, a highly liquid company, the stock exchange index appreciated by 124.3 %. Every stock market operates in an economy and hence, happenings in the economy affect the performance of the market. For the Ghana stock market, the main factor which significantly affects its performance is the general elections held after every 4 years. As can be noticed from Fig. 1, the stock market becomes volatile during the periods after the general elections, as in 2000–2001, and 2008–2009. Also during these elections, there were regime changes which may have concerned investors in the market thereby influencing their decisions. This contributed to the volatilities observed during those periods.

Since the financial market usually only provides the raw data of the realized values of the various financial indices, the daily log-returns, which for the purpose of this paper, will be used interchangeably with returns, were derived as follows

18 $$r_{t} = ln\left( D_t / D_{t-1} \right) \times 100$$

where $r_{t}$ denotes the daily logarithmic return at day *t*, $D_{t}$ represents the daily return at day *t* and $ln$ represents the natural logarithm.

## Results and discussions

This paper applies the extreme value theory (EVT) approach in analysing extreme returns of the Ghana stock exchange all-shares index over the period 2000–2010. An in-depth analysis of the extreme value methodology applied to the high frequency (daily) Ghana stock data is presented in this section. The yearly progression of the data is illustrated by means of logarithmic time series plot. Furthermore, the volatility of the financial returns over the period is examined. The establishment of the presumption of financial returns having fat tails is made from an examination of the histogram of returns. Various risk measures are then computed and discussed.

Table 1 of Appendix presents a descriptive statistics of the data. It shows that the data has a positive mean value and also exhibits strong positive skewness which indicates that the bulk of the data resides in the right tail of the distribution of the data which most likely means that the right tail is more extreme. The series further revealed a high kurtosis value of 40.16923, far in excess of the normal distribution value of 3. This is evidence of the fat tailed nature of the distribution of the returns data. The Jarque–Bera test for normality resulted in a p value of 0.000 hence rejecting the hypothesis of the data being normally distributed. This is however not surprising considering the magnitude of the skewness and kurtosis values.

**Fig. 2:**
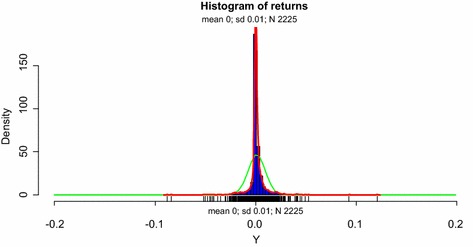
Histogram of returns of daily GSE all-shares index

Figure 2 illustrates the histogram of the daily returns data. The red curve represents the empirical density function of the returns distribution whilst the green curve depicts the normal density. It can be observed that the empirical distribution function shows a very high peak around its mean and also relatively fatter tails compared to the normal. This conforms to the conclusion of the data deviating from normality established above. The histogram also shows that relatively more observations lie to the right of the mean of the distribution, compared to the left. This is also in agreement of the positive skewness obtained and the high peak corresponds to the large kurtosis value obtained.

Figure 1 presents a plot of the log returns of the daily GSE all-shares. It shows clearly that the Ghana stock market experienced some periods of high volatility and other periods of relatively stable movement as explained earlier. Also, volatility clusters can be observed where periods of high or low changes in the returns are accompanied by other high or low changes.

Furthermore, Phillips-Perron and Augmented Dickey-Fuller unit root tests were performed to check for stationarity and it was found that the returns data is fairly stationary.

In order to apply the Extreme Value method to any data, it is a strong requirement for the data to be independent and identically distributed (iid). Hence, a Box-Ljung (lag = 8) test for autocorrelation was performed. The test revealed the presence of significant autocorrelation in the returns series. Furthermore, a Lagrangian Multiplier (LM) test performed to examine the data for the presence of autoregressive conditional heteroscedastic (ARCH) effects also indicated the presence of significant ARCH effects in the data ($$ \chi^{2} = 90.6534, \;df = 12, $$$$ p = 0.000 $$).

As recommended by McNeil and Frey (2000), to produce a complete iid process with relatively no autocorrelation terms and no heteroscedastic effects, different combinations of ARMA-GARCH models were fitted and based on the AIC and BIC values, the ARMA (1, 1)-GARCH (1, 1) model was found to be the best fitting model (model parameters presented in Table 2 of Appendix). Consequently, the residuals from the ARMA (1, 1)-GARCH (1, 1) model were extracted with their corresponding conditional variances. A standardized independent and identically distributed series was then calculated as $r_{t} = \frac{{e_{t} }}{{\sigma_{t} }}$, where $e_{t}$ is the residual term at a time *t* and $\sigma_{t}$ is the corresponding conditional standard deviation.

Autocorrelation and conditional heteroscedasticity tests performed on the standardized series and squared standardized series showed that no autocorrelation existed and also no persistence of variance and hence no evidence of volatility clustering in the series. Also, there were no conditional heteroscedastic terms in the standardized series.

The standardized series was therefore considered suitable for the application of the extreme value analysis.

The first step in the application of the Peak Over Threshold (POT) approach of the EVT is the selection of appropriate threshold levels for the tails of the distribution. This was performed graphically in this study by the use of hill plots, shape parameter plots and mean excess plots.

**Fig. 3:**
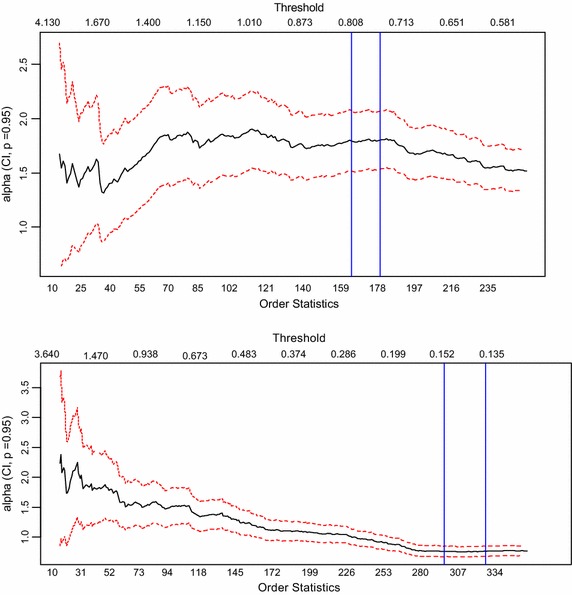
Hill Plots (*right tail* on the *top*, *left tail* on the *bottom*)

Figure 3 shows the hill plots for the tails of the standardized series. For the right tail, the last 250 order statistics are plotted and the last 350 plotted for the left tail, in each case leaving more than 10 % of the data for the analysis. This is considered in practice to be a fair compromise. Interest here was in determining a relatively steady area on the graph where the order statistics obtained under the area is sufficiently large such that thresholds selected in that area will also be relatively steady and provide sufficient exceedances to be fitted by the GPD. For the plot of the right tail, such an area was determined to be between 160 and 175 order statistics and in the case of the left tail, the area was determined to be between 290 and 320 (in both cases indicated by the blue vertical lines on the plot). Hence, a sufficient threshold was expected to lie in these ranges.

**Fig. 4:**
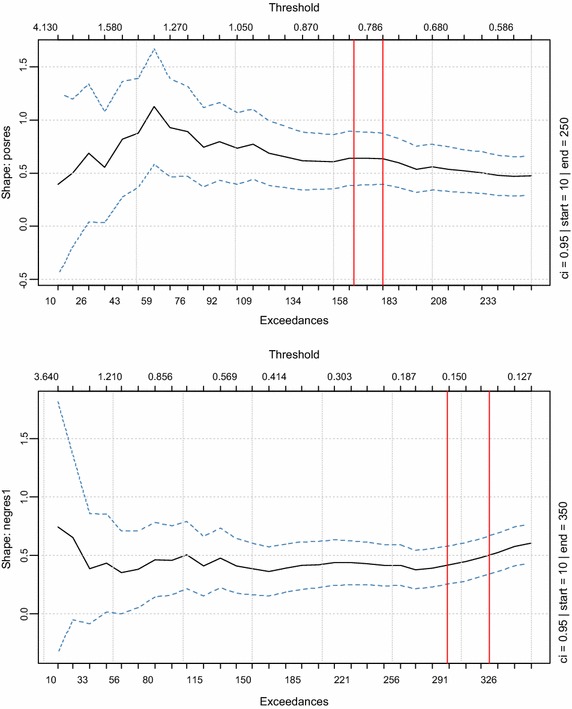
GPD shape parameter plots (*right tail* on the *top*, *left tail* on the *bottom*)

Upon examination of the shape parameter plots in Fig. 4, it was observed that in both cases the graphs are quite steady in the ranges determined by the hill plots. Thus the shape parameters are considered to be stable within these ranges (also indicated by red vertical lines on the plot). The corresponding thresholds determined for these ranges were (0.13–0.165) for the left tail and (0.786–0.83) for the right tail.

**Fig. 5:**
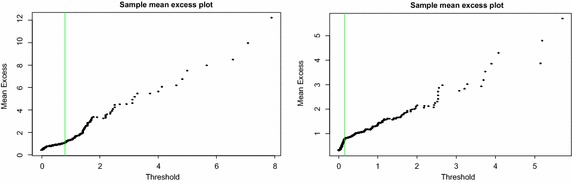
Mean excess (ME) plots (*right tail* on the *left*, *left tail* on the *right*)

Finally, Fig. 5 shows the mean excess plots. Based on the two previous graphing techniques considered, the thresholds are expected to lie in some specified ranges. Upon examining closely the graphs of the mean excess plots, a threshold value of *u* = 0.8 is selected for the right tail and *u* = 0.15 for the left tail. These points are represented by the faint green vertical lines on the graphs. It can be noticed that in both cases, the graphs are relatively stable up to the points selected and from there a slight kink is observed above which the graphs exhibit slight variations even though the slopes remain positive. Furthermore, these selected thresholds fall in the ranges determined by the hill plots and shape parameter plots.

There were 169 observations above the selected threshold left for modelling for the right tail and 303 observations left above the threshold in the left tail. These were considered enough for a GPD fit since they are more than 10 % of the total observations in each of the tails. It can be also seen that for the threshold selected within the ranges indicated, the curves above the threshold have an upward slope and will therefore be well approximated by the Generalized Pareto Distribution (GPD) with positive shape parameters.

Table 3 of Appendix presents the results obtained from fitting the Generalized Pareto Distribution (GPD) to the tails of the standardized series. It was observed that the Maximum Likelihood Estimates (MLE) of the GPD fitted to the left tail had standard errors of 0.0856 for the shape parameter and 0.04323 for the scale parameter, which are smaller compared to that of the Probability Weighted Moments (PWM) estimates of 0.12064 and 0.04863 respectively. However, for the right tail, the asymptotic standard errors were not available for the PWM shape parameter estimate since the shape parameter is greater than 0.5 $(\hat{\xi } > 0.5)$ as indicated by Rootzen and Tajvidi (1997). The standard error of the scale parameter estimate was also smaller for the MLE compared to the PWM method. The MLE estimates therefore fitted the data better since they provide smaller standard errors in estimation. Finally, it was also observed that the MLE estimates’ standard error for the right-tail parameters estimate were greater compared to the left-tail. This indicated that the distribution provided a better fit to the left-tail compared to the right.

### Model diagnostics

**Fig. 6:**
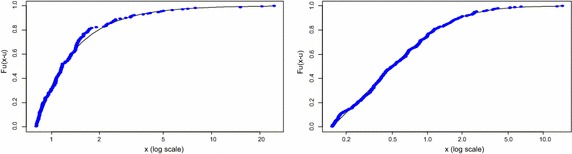
Plots of fitted GPD model (*right tail* on the *left*, *left tail* on the *right*)

Figure 6 illustrates a plot of the estimated GPD models fitted as curves against the empirical excesses over the selected thresholds, with the right tail fit on the left and the left tail fit on the right. Both plots show that the estimated GPD models provide a very good fit to the extreme values since all the points on the plots lie approximately on the curve.

**Fig. 7:**
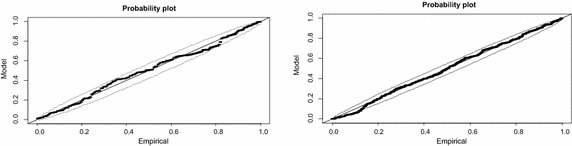
PP plots of fitted GPD model (*right tail* on the *left*, *left tail* on the *right*)

The Probability (PP) plots of the goodness of fit of the GPD models on the empirical excesses are presented in Fig. 7. It can be seen that for both graphs, the plotted points all lie inside the confidence bands. Hence the models fit quite well for both the right and left tails. However, it can be observed that the plot of the right tail fit (shown on the right) indicates more departures from the straight line as compared to the plot of the left tail fit. It can be inferred from this that although both models provide good fits, the GPD model of the left tail excesses fits quite better than the right tail fit.

**Fig. 8:**
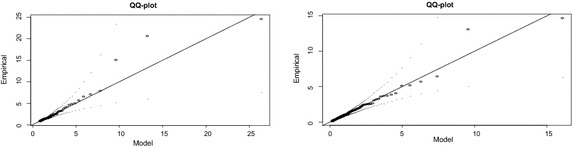
QQ plots of fitted GPD model (*right tail* on the *left*, *left* tail on the *right*)

The results obtained by fitting the empirical quantiles of excesses against the quantiles of the fitted GPD models in quantile–quantile (Q–Q) plots are displayed in Fig. 8. The plots show that for both tails, the points of the exceedances do not deviate significantly from the straight line and also they all fall within the confidence bands. The points of the largest observations can be observed on the plots but are not considered to be very significant departures from the fitted models since they still fall within the confidence bands and are also not very distant from the straight line.

**Fig. 9:**
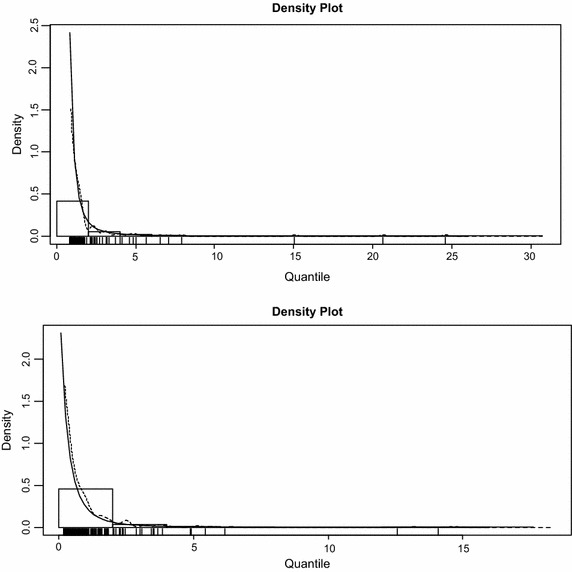
Density plots of fitted GPD model (*right tail* on the *top*, *left tail* on the *bottom*)

The graphs of the density plots exhibited in Fig. 9 confirm the results obtained from the PP plots. They show that the GPD models provide adequate fits to the exceedances but the model for the left tail provides a better fit since most of the points lie on the curve of the GPD distribution as compared to the right tail which shows a few departures from the curve.

**Fig. 10:**
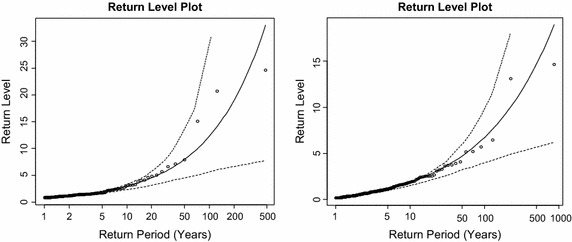
Return level plots of fitted GPD model (*Right tail* on the *left*, *left tail* on the *right*)

Figure 10 shows the return level plots of the right and left tails. The return level plot presents a graph of the empirical estimates of the return level function plotted against the estimated return levels from the fitted model. For diagnostic purposes, a model is deemed desirable if there are no significant departures from the curve or if there exists no points outside the confidence bands located above and below the curve. It can be observed from the plots for both tails that all the points lie approximately on the line and no points can be found outside the confidence bands.

### Risk measures

Table 4 of Appendix presents the results obtained after computing the risk measures associated with both tails after fitting the extreme value distribution. The results indicate that with a probability of 0.05, thus 95 % level of confidence, the expected market return would not gain by more than 1.39 % and if it does increase by more than 1.39 %, an average gain of 3.55 % is expected within a one-day duration. Analogously, the daily loss will not exceed 1.34 % with a probability of 0.05 and the expected loss if it does exceed this level is 3.08 %.

For the higher quantiles, the results show that with a probability of 0.005, i.e. 99.5 % level of confidence, the daily market gains will not exceed 5.51 %. The expected daily market gains if the level 5.51 % is exceeded is 14.794 %. Similarly, the expected daily market losses will not exceed 5.215 % at a probability of 0.005 and an expected loss of 10.062 % is obtained if the losses exceed that level. Furthermore, at 99.9 % level of confidence i.e. probability of 0.001, the daily market gains will not exceed 15.021 % and if it does exceed that level, the expected daily market gain is 40.773 %. On the other hand, the daily market losses will not exceed 11.54 % with an expected daily market loss of 21.464 % if the losses do exceed 11.54 %.

Alternatively, the results above may be interpreted in terms of holding an investment position (short or long position) on the stock market. For the lower quantile, the daily loss associated with holding a long position on the market is at most 1.34 % with a probability of 0.05 and in the case where it is exceeded, a loss of 3.08 % is expected. Conversely, the daily gain associated with holding a short position on the market will not exceed 1.39 % with a corresponding expected gain of 3.55 % in the situation where the gain exceeds 1.39 %. For the higher quantile, with a probability of 0.001 (i.e. 99.9 % level of confidence), a holder of a long position may experience daily losses not in excess of 11.54 % and if the losses do exceed 11.54 %, the expected losses will be 21.46 %, and the holder of a short position may experience a daily gain not exceeding 15.02 % with a probability of 0.001 and in the event it does, the expected gain observed will be 40.77 %.

This paper employs the VaR in determining the best and worst case scenarios of the market value of the Ghana stock exchange all-shares index over one trading day, at a given level of confidence. Hence the above results imply that for an investment of $1 million in the market, the expected gains will not exceed $13,900, with a 95 % confidence level, over one trading day. On the other hand, the worst daily loss on the investment will not exceed $13,400. Also, with a 99.9 % level of confidence, a $1 million investment in the market will yield a daily gain not in excess of $150,200 and a daily loss not exceeding $115,400.

These results indicate that for an investment in the Ghana stock market, the possibility of losses is lower than the possibility of gains. This is in contrast with findings from the work of Gilli and Kellezi (2006) who found that for the Hang Seng (HS), DJ Euro Stoxx 50 (ES50), Nikkei, Swiss Market Index (SMI), and FTSE100 market indices, the exposure to extreme losses is higher than the possibility of extreme gains. However, the S&P500 stock index was found to be more exposed to extreme gains than extreme loses, hence consistent with the findings of this paper.

## Conclusion

The main aim of the paper was to empirically examine the application of the EVT methodology in the Ghanaian stock market by applying it to the Ghana stock exchange all-shares indices. The results of the study showed that the daily returns of the Ghana stock exchange all-shares index data was from a distribution with fat-tails and asymmetric in nature and hence the extreme value (EVT) model provided a better fit to the tails of the distribution of returns. As a result of the observed volatility in the daily returns data, the conditional EVT approach was preferred for the study. A similar realization was made by Polakow and Seymour (2003) when they compared the conditional and unconditional approaches in the modelling of a volatile South African stock market, and the conditional approach provided better results compared to the unconditional approach. The paper considered the Peak Over Threshold (POT) method of the EVT approach, which fitted a Generalized Pareto Distribution (GPD) to excesses above thresholds $u = 0.8$ and $u = 0.15$ for the right and left tails respectively. Among the two methods considered for estimating the parameters of the GPD, the maximum likelihood estimation (MLE) method was shown by the study to provide more accurate estimates for both tails with lower standard errors compared to the method of probability weighted moment (PWM) estimation. However, the standard error for the right-tail parameter estimates were observed to be greater compared to the left-tail. This was further observed in the diagnostic plots where the right tail fit (shown on the right) indicated more departures from the straight line as compared to the plot of the left tail fit. It can be inferred from this that although both models provide good fits, the GPD model of the left tail of the Ghana stock returns excesses fits quite better than the right tail fit.

Andjelic et al. (2010) concluded that in emerging markets, different characteristics are observed at each of the tails of the return distributions which indicates that risk and reward are not equally likely in these markets. Bi and Giles (2007) however concluded that the GPD performs very well in modelling both the positive and negative returns of the tails distributions.

It was further indicated by Gencay and Selcuk (2004) that EVT based VaR estimates were more accurate at higher quantiles. Moreover, they reveal that the different daily return distributions have different moment properties at their right and left tails, and as some studies, including Krehbiel and Adkins (2005) concluded, the upper and lower tails behave differently, and thus should be treated separately while estimating risk measures.

Consequently, value at risk (VaR) and expected shortfall (ES) risk measures were computed at some high quantiles from incorporating the GPD models of the tails of the distribution of daily Ghanaian stock returns. They revealed that in the Ghanaian stock market, the VaR and ES associated with the left tail or long investment position increased more significantly as the quantiles increased as compared to the right tail or short investment position, except for the last quantile (99.9th) for which the right tail had a more significant increase in the VaR and ES.

Quismorio (2010) indicated that emerging markets usually have fatter negative tails compared to developed markets and hence financial crashes are more likely in emerging markets. This paper attributes this to the fragile political environment and unstable macroeconomic situation in Ghana and most emerging markets. This therefore implies that risk managers and investors may need to allocate substantial capital to compensate for possible losses in extreme situations since such losses may be quite massive.

To conclude, this study revealed that the Peak over Threshold approach of the extreme value theory, which fits a Generalized Pareto Distribution to extremes above a certain threshold, can be very efficient in the modeling of extreme events and assessing the size of potential extreme risks, particularly in the stock market.

## Appendix

See Tables 1, 2, 3 and 4.

**Table 1:** Descriptive statistics

GSE returns series	Value
Mean	0.001071323
Maximum	0.1211834
Minimum	−0.08813781
Standard deviation	0.00851636
Skewness	0.9737527
Kurtosis	40.16923
Jarque–Bera Test	149,942.7
(Probability)	0.0000
Number of observations	2225

**Table 2:** Results from ARMA (1, 1)-GARCH (1, 1) model

Parameter	Estimate	Standard error	P value
AR (1)	0.8986	0.01562	0.000
MA (1)	−0.8437	0.02327	0.000
$\omega$	6.405 × 10^−8^	0.918	0.3587
$\alpha$	0.1204	0.1797	0.000
$\beta$	0.7912	0.03566	0.000

**Table 3:** Results from fitting the GPD model

Estimate	MLE		PWM	
	Right tail	Left tail	Right tail	Left tail
	$u = 0.8$	$u = 0.15$	$u = 0.8$	$u = 0.15$
Shape parameter $(\hat{\xi })$	0.6339	0.4455	0.6147	0.43
Conf. interval (lower, upper)	(0.3968, 0.8702)	(0.2777, 0.6133)	(0.2871, 0.7994)	(0.2647, 0.6021)
Std. error	0.12097	0.0856	–	0.12064
Scale parameter $(\hat{\sigma })$	0.4142	0.4329	0.4153	0.4348
Conf. interval (lower, upper)	(0.3048, 0.5237)	(0.3482, 0.5177)	(0.3139, 0.5622)	(0.3492, 0.5201)
Std. error	0.05584	0.04323	0.1306	0.04863

**Table 4:** Risk measures (VaR and ES are in percentages)

Probability	Value at risk (VaR)	Expected shortfall (ES)
Measures of risk—right tail distribution (GPD fit)		
0.950	1.392526	3.549663
0.975	2.079958	5.427241
0.990	3.602491	9.585723
0.995	5.509216	14.793548
0.999	15.020921	40.772793
Measures of risk—left tail distribution (GPD fit)		
0.950	1.3429886	3.081368
0.975	2.1260213	4.492982
0.990	3.6116033	7.171118
0.995	5.2149448	10.061545
0.999	11.5398391	21.463761
